# Sensitivity of ICD coding for sepsis in children—a population-based study

**DOI:** 10.1007/s44253-023-00006-1

**Published:** 2023-06-13

**Authors:** Olga Endrich, Karen Triep, Luregn J. Schlapbach, Klara M. Posfay-Barbe, Ulrich Heininger, Eric Giannoni, Martin Stocker, Anita Niederer-Loher, Christian R. Kahlert, Giancarlo Natalucci, Christa Relly, Thomas Riedel, Christoph Aebi, Christoph Berger, Philipp K. A. Agyeman, on behalf of the Swiss Pediatric Sepsis Study, Luregn J. Schlapbach, Klara M. Posfay-Barbe, Ulrich Heininger, Eric Giannoni, Martin Stocker, Anita Niederer-Loher, Christian R. Kahlert, Giancarlo Natalucci, Christa Relly, Thomas Riedel, Christoph Aebi, Christoph Berger, Philipp K. A. Agyeman, Walter Bär, Sara Bernhard-Stirnemann, Paul Hasters, Gabriel Konetzny, Antonio Leone

**Affiliations:** 1 Department of Clinical Chemistry, Inselspital, Bern University Hospital, University of Bern, Bern, Switzerland; 2 Medical Directorate, Inselspital, Bern University Hospital, University of Bern, Bern, Switzerland; 3 Department of Intensive Care and Neonatology, and Children’s Research Center, University Children’s Hospital Zurich, Zurich, Switzerland; 4 Child Health Research Center, University of Queensland, Brisbane, Australia; 5 Pediatric Infectious Diseases Unit, Children’s Hospital of Geneva, University Hospitals of Geneva, Geneva, Switzerland; 6 Infectious Diseases and Vaccinology, University of Basel Children’s Hospital, Basel, Switzerland; 7 Clinic of Neonatology, Department Mother-Woman-Child, Lausanne University Hospital and University of Lausanne, Lausanne, Switzerland; 8 Children’s Hospital Lucerne, Lucerne, Switzerland; 9 Children’s Hospital of Eastern Switzerland St. Gallen, St. Gallen, Switzerland; 10 Family Larsson-Rosenquist Foundation Centre for Neurodevelopment, Growth and Nutrition of the Newborn, Department of Neonatology, University Hospital Zurich, Zurich, Switzerland; 11 Division of Infectious Diseases and Hospital Epidemiology, Children’s Research Center, University Children’s Hospital Zurich, Zurich, Switzerland; 12 Department of Pediatrics, Inselspital, Bern University Hospital, University of Bern, Bern, Switzerland; 13 Department of Pediatrics, Cantonal Hospital Graubuenden, Chur, Switzerland

**Keywords:** Bacteremia, International Classification of Diseases, Systemic inflammatory response syndrome, Population surveillance, Clinical coding, Critical care

## Abstract

**Background:**

International Classification of Diseases 10th edition (ICD-10) is widely used to describe the burden of disease.

**Aim:**

To describe how well ICD-10 coding captures sepsis in children admitted to the hospital with blood culture-proven bacterial or fungal infection and systemic inflammatory response syndrome.

**Methods:**

Secondary analysis of a population-based, multicenter, prospective cohort study on children with blood culture-proven sepsis of nine tertiary pediatric hospitals in Switzerland. We compared the agreement of validated study data on sepsis criteria with ICD-10 coding abstraction obtained at the participating hospitals.

**Results:**

We analyzed 998 hospital admissions of children with blood culture-proven sepsis. The sensitivity of ICD-10 coding abstraction was 60% (95%-CI 57–63) for sepsis; 35% (95%-CI 31–39) for sepsis with organ dysfunction, using an explicit abstraction strategy; and 65% (95%-CI 61–69) using an implicit abstraction strategy. For septic shock, the sensitivity of ICD-10 coding abstraction was 43% (95%-CI 37–50). Agreement of ICD-10 coding abstraction with validated study data varied by the underlying infection type and disease severity (*p* < 0.05). The estimated national incidence of sepsis, inferred from ICD-10 coding abstraction, was 12.5 per 100,000 children (95%-CI 11.7–13.5) and 21.0 per 100,000 children (95%-CI 19.8–22.2) using validated study data.

**Conclusions:**

In this population-based study, we found a poor representation of sepsis and sepsis with organ dysfunction by ICD-10 coding abstraction in children with blood culture-proven sepsis when compared against a prospective validated research dataset. Sepsis estimates in children based on ICD-10 coding may thus severely underestimate the true prevalence of the disease.

**Supplementary Information:**

The online version contains supplementary material available at 10.1007/s44253-023-00006-1.

## Introduction

As evidenced by the COVID-19 pandemic, effective responses of healthcare systems rely on robust surveillance systems and databases. For sepsis, one of the most common diseases causing morbidity and mortality globally, most countries do not have a surveillance system or national database. Surveillance is often done indirectly using the International Statistical Classification of Diseases and Related Health Problems (ICD) coding [1, 2]. In a recent meta-analysis of the global burden of sepsis in children, 5 of 9 studies were based on ICD case identification [3].

Infections remain one of the leading causes of death in childhood [4]. Sepsis, defined as life-threatening organ dysfunction caused by a dysregulated host response to infection [5], is a common final pathway of many infections leading to death [6, 7]. Children, especially those less than 5 years old, are disproportionately affected by sepsis, and neonatal and pediatric age groups are estimated to account for 50% of global sepsis cases [2]. The World Health Organization (WHO) has declared sepsis a global public health priority and identified the correct application of the ICD system as one of the key steps towards better prevention, diagnosis, and care for sepsis [8]. Problems with inaccurate representation of sepsis by ICD have long been reported in adults [9, 10], and also confirmed for the 10th revision of ICD (ICD-10) [10]. In most of these studies, the reference standard against which ICD codes were compared was other databases or retrospective chart reviews [9, 10]. Only few studies have used prospective databases for the verification of the accuracy of ICD-based sepsis abstraction [9, 11], and there is a lack of pediatric data assessing the accuracy of ICD sepsis coding [11, 12].

We aimed to assess the accuracy of ICD coding abstraction for the diagnosis of sepsis in children using data from the Swiss Pediatric Sepsis Study, a prospective, population-based cohort study on blood culture-proven sepsis in children [13]. We hypothesized that surveillance based on sepsis coding in ICD-10 leads to underreporting of sepsis and sepsis with organ dysfunction.

## Methods

### Study design

The Swiss Pediatric Sepsis Study, a multicenter prospective cohort study in ten pediatric centers in Switzerland, investigated the epidemiology of sepsis in children from November 2011 to December 2015 [13]. The study consecutively included neonates and children younger than 17 years with blood culture-proven invasive bacterial or fungal infection, if they met the systemic inflammatory response syndrome (SIRS) criteria according to the 2005 International Pediatric Sepsis Consensus Conference (IPSCC) definitions [14]. Investigators prospectively assessed the SIRS criteria. During the study period, the study accounted for 78% of hospital and 98% of intensive care unit (ICU) admissions in children with an invasive bacterial or fungal infection in Switzerland [13]. Children after allogeneic bone marrow transplantation and those with contaminated blood cultures were excluded [13].

For this secondary analysis, we only included data on sepsis episodes with full information on organ dysfunction and if ICD-10 discharge codes were available. We followed the Standards for Reporting Diagnostic Accuracy statement [15].

### ICD-10 coding

During the study years, ICD-10 with German modification codes was used to encode 1 main and up to 49 secondary diagnoses of every hospital stay in the medical statistic dataset of participating hospitals. All hospitals in Switzerland must submit this data to the Federal Office of Statistics annually [16]. ICD coding is performed by coding experts and governed by guidelines that are revised annually by the Swiss Federal Office of Statistics. ICD coding guidelines for sepsis in Switzerland evolved from Sepsis-1 to Sepsis-2 Consensus definitions in 2014. The 2005 IPSCC definitions for sepsis in children were adopted in 2015. For neonates, depending on postnatal age at sepsis onset, codes from chapter XVI on Perinatal Conditions (P00—P96) apply. Encoding is based on discharge letters and additional hospital documents. The SwissDRG performs annual audits of hospital coding data through its case mix office to prevent upcoding and ensure consistency of coding. We extracted ICD-10 information on hospital admissions with sepsis episodes included in the Swiss Pediatric Sepsis Study from the medical statistics dataset of participating hospitals.

### Definition of sepsis and organ dysfunctions

#### Swiss pediatric sepsis study

All children included in the Swiss Pediatric Sepsis Study experienced sepsis as defined by the 2005 IPSCC, and investigators prospectively assigned organ dysfunctions according to the 2005 IPSCC criteria [14, 17, 18]. The study team additionally monitored organ dysfunctions using all available clinical and laboratory investigations performed during the sepsis episode. In patients experiencing more than one sepsis episode during the same hospital stay, we took the worst status for each organ dysfunction to define the numbers of organ dysfunctions present during the entire hospital stay.

#### ICD-10 coding

For the ICD-10 coding abstraction of bacterial or fungal infection, we used pathogen-specific sepsis codes, pathogen-specific codes not associated with sepsis, and unspecific codes for bacterial infections (Additional file 1). For the ICD-10 coding abstraction of sepsis, we used the combination of ICD-10 codes for bacterial and fungal infection and SIRS codes (R57.2, R65.0, R65.1) and/or septic shock (A483). For the ICD-10 coding abstraction of sepsis with organ dysfunction, we compared an “explicit” coding strategy—using a combination of ICD-10 codes for bacterial or fungal infection with ICD-10 codes specifically designed to capture SIRS and sepsis with organ dysfunction (R57.2, R65.1, and A48.3)—with an “implicit” coding strategy, additionally including a selection of ICD-10 codes for specific organ dysfunctions (Additional file 1).

### Statistical analysis

We have presented descriptive statistics of continuous data as median with interquartile range (IQR) and of categorical data as frequency with percentage. We calculated sensitivity, specificity, accuracy, positive and negative predictive values, and positive and negative likelihood ratios of explicit and implicit ICD-10 coding abstraction for sepsis with organ dysfunction using the validated Swiss Pediatric Sepsis Study data as a reference standard. For binomial proportions and likelihood ratios, we calculated corresponding 95% confidence intervals (CIs) [19, 20].

To explore the agreement of ICD-10 coding abstraction with validated study data in different subgroups of the Swiss Pediatric Sepsis Study population, we fitted multivariate binomial regression models. We defined agreement strictly as concordance with the validated study data with one exception: For implicit ICD-10 coding abstraction, we allowed for the absence of organ dysfunction in the validated study data because organ dysfunction might have had other reasons than sepsis. We included the patient risk category, acquisition of the infection in the hospital, presence of an organ dysfunction, ICU admission, pathogens, site of infection, and death as explanatory variables. To correct for the correlation between multiple observations at the same hospital, we used a random effect. We have presented the results of regression analysis as odds ratios (ORs) with 95%-CIs and *p* values based on likelihood ratio tests.

We calculated the incidence of blood culture-proven bacterial sepsis for the study years 2012–2015. To calculate age-specific incidence, we divided the number of annual admissions with sepsis in the study by the end-of-year resident population in Switzerland in the respective age groups [21]. We calculated the incidence for different age groups, age-standardized to the European standard population, and estimated the national incidence as previously described [13].

We considered a two-sided *p* value less than 0.05 significant. We did all analyses and plots with R version 4.2.2 [22].

## Results

During the 52-month study period, 1005 episodes of blood culture-proven sepsis in 998 hospital admissions met the eligibility criteria (Additional file 1: Fig. S1). Three hundred nineteen (32%) hospital admissions were in previously healthy children, 343 (34%) in neonates, and 336 (34%) in children with comorbidities. The median age at blood culture sampling was 8 months (IQR 0.6–68 months), and 401 (40%) of the admitted children were girls. In 362 (36%) admissions, sepsis was hospital-acquired, and in 507 (51%), children were cared for in a neonatal or pediatric ICU during sepsis. In 572 (57%) admissions, at least one sepsis-related organ dysfunction was present. Sixty-seven (7%) children died in the first 30 days after blood culture sampling, with a 30-day case fatality ratio in sepsis with organ dysfunction of 12% (66 of 572, Table 1).

**Table 1:** Demographic and clinical characteristics of children with blood culture-proven sepsis

	All hospital admissions in children with sepsis, *n* = 998	Hospital admissions without the agreement of ICD-10 coding abstraction of sepsis with validated study data, *n* = 402	Hospital admissions with the agreement of ICD-10 coding abstraction of sepsis with validated study data, *n* = 596
Median age at sepsis onset [years (IQR)]	0.67 (0.05–5.71)	2.47 (0.09–7.60)	0.26 (0.03–3.62)
Age groups			
Preterm newborn	225 (23%)	70 (17%)	155 (26%)
Term newborn (< 28 days)	118 (12%)	33 (8%)	85 (14%)
28–365 days	186 (19%)	58 (14%)	128 (21%)
1–4 years	198 (20%)	94 (23%)	104 (17%)
5–9 years	131 (13%)	76 (19%)	55 (9%)
10–16 years	140 (14%)	71 (18%)	69 (12%)
Sex			
Female	401 (40%)	161 (40%)	240 (40%)
Male	597 (60%)	241 (60%)	356 (60%)
Ethnicity			
White European	771 (77%)	310 (77%)	461 (77%)
Asian	32 (3%)	17 (4%)	15 (3%)
African	48 (5%)	21 (5%)	27 (5%)
Mixed	44 (4%)	13 (3%)	31 (5%)
Others	17 (2%)	8 (2%)	9 (2%)
Missing	86 (9%)	33 (8%)	53 (9%)
Type of sepsis acquisition			
Community-acquired	636 (64%)	269 (67%)	367 (62%)
Hospital-acquired	362 (36%)	133 (33%)	229 (38%)
CVAD present at the time of sepsis onset			
No	626 (63%)	241 (60%)	385 (65%)
Yes	372 (37%)	161 (40%)	211 (35%)
Median length of hospital stay [days (IQR)]	16 (9–44)	14 (7–34)	17 (10–52)
Median length of hospital stay after sepsis onset [days (IQR)]	14 (8– 32)	12 (7–26)	15 (10–38)
Median length of antibiotic treatment for sepsis [days (IQR)]	14 (10–16)	14 (10–21)	14 (10–15)
Number of organ dysfunctions			
None	426 (43%)	209 (52%)	217 (36%)
One	282 (28%)	116 (29%)	166 (28%)
Two	119 (12%)	39 (10%)	80 (13%)
Three	74 (7%)	17 (4%)	57 (10%)
Four	52 (5%)	13 (3%)	39 (7%)
Five	31 (3%)	4 (1%)	27 (5%)
Six	14 (1%)	4 (1%)	10 (2%)
ICU admission			
No	491 (49%)	262 (65%)	229 (38%)
Yes	507 (51%)	140 (35%)	367 (62%)
Reason for ICU admission			
Already on ICU at sepsis onset	256 (26%)	78 (19%)	178 (30%)
Admitted to ICU for sepsis	251 (25%)	62 (15%)	189 (32%)
Median length of ICU stay [days (IQR)]	15 (4–52)	22 (5–66)	13 (4–48)
Median length of ICU stay after sepsis onset [days (IQR)]	10 (3–36)	12 (4–48)	10 (3–32)
Non-invasive ventilation			
No non-invasive ventilation	843 (84%)	352 (88%)	491 (82%)
Already on non-invasive ventilation at sepsis onset	95 (10%)	33 (8%)	62 (10%)
Non-invasive ventilation due to sepsis	60 (6%)	17 (4%)	43 (7%)
Invasive ventilation			
No invasive ventilation	713 (71%)	324 (81%)	389 (65%)
Already on invasive ventilation at sepsis onset	91 (9%)	31 (8%)	60 (10%)
Invasive ventilation due to sepsis	194 (19%)	47 (12%)	147 (25%)
Catecholamine administration			
No	833 (83%)	370 (92%)	463 (78%)
Yes	165 (17%)	32 (8%)	133 (22%)
Case fatality			
Survived	931 (93%)	385 (96%)	546 (92%)
Died	67 (7%)	17 (4%)	50 (8%)
Median time to death from sepsis onset [days (IQR)]	3 (0–11)	4 (0–7)	2 (1–12)

Data are shown separately for hospital admissions without and with the agreement of ICD-10 coding abstraction with validated study data

*CVAD* central venous access device, *ICD* International Statistical Classification of Diseases and Related Health Problems, *IQR* interquartile range, *ICU* intensive care unit

### Sensitivity of ICD-10 coding abstraction for bacteremia and sepsis compared to validated study data

A total of 895 (90%, 95%-CI 88–91) admissions were assigned an ICD-10 code for a bacterial or fungal infection, with a correct representation of the causative pathogen in 832 (83%, 95%-CI 81–86). A total of 596 (60%, 95%-CI 57–63) admissions, including 379 (66%, 95%-CI 62–70) admissions with research-confirmed organ dysfunction, were assigned an ICD-10 code for sepsis. The sensitivity of ICD-10 coding abstraction for sepsis increased from 53% (95%-CI 47–60) in 2012 to 65% (95%-CI 59–70) in 2015 (*p* = 0.01). Based on ICD-10 coding abstraction, the 30-day case fatality ratio in children with sepsis was 8% (95%-CI 6–11) (50 of 596), although 25% (17 of 67) deaths in children with sepsis were not captured. The sensitivity of the ICD-10 coding abstraction of bacterial and fungal infections varied by pathogen and was better in children admitted to the ICU (Additional file 1: Table S1). The sensitivity of ICD-10 coding abstraction of sepsis varied by pathogen and by site of infection and was better in children admitted to the ICU (Additional file 1: Table S2).

### Agreement of ICD-10 coding abstraction of sepsis with organ dysfunction with validated study data

A total of 221 (39%) admissions with research-confirmed sepsis with organ dysfunction had been assigned explicit ICD-10 codes for sepsis with organ dysfunction with an accuracy of 60% (95%-CI 57–63) (Table 2). The sensitivity of explicit ICD-10 coding abstraction of sepsis with organ dysfunction increased from 31% (95%-CI 23–39) in 2012 to 38% (95%-CI 31–46) in 2015 (*p* = 0.2). In hospital admissions assigned explicit ICD-10 codes for sepsis with organ dysfunction, the case fatality ratio was 18% (95%-CI 14–24) (40 of 221) although 39% (26 of 66) deaths in children with sepsis with organ dysfunction were not captured. The agreement of explicit ICD-10 coding abstraction with validated study data varied by site of infection, pathogen and risk category and was better in children admitted to ICU and in children who did not survive (Table 3).

**Table 2:** Sensitivity and specificity of ICD-10 coding abstraction for sepsis-related organ dysfunction

	Any sepsis-associated organ dysfunction present according to validated study data	No sepsis-associated organ dysfunction present according to validated study data	Sensitivity [% (95%-CI)]	Specificity [% (95%-CI)]	Positive predictive value [% (95%-CI)]	Negative predictive value [% (95%-CI)]	Positive likelihood ratio (95%-CI)	Negative likelihood ratio (95%-CI)
Explicit ICD-10 coding abstraction			35 (31–39)	95 (92–97)	90 (85–93)	52 (48–55)	6.7 (4.4–10.3)	0.69 (0.65–0.73)
Sepsis with organ dysfunction	199	22						
No sepsis with organ dysfunction	373	404						
Implicit ICD-10 coding abstraction			65 (61–69)	87 (84–90)	87 (84–90)	65 (61–69)	5 (3.9–6.5)	0.4 (0.36–0.45)
Sepsis with organ dysfunction	371	55						
No sepsis with organ dysfunction	201	371						

*CI* confidence interval, *ICD* International Statistical Classification of Diseases and Related Health Problems

**Table 3:** Determinants of agreement of ICD-10 coding abstraction of sepsis with organ dysfunction with validated study data

	All hospital admissions in children with sepsis, *n* = 998	Explicit ICD-10 coding abstraction of sepsis with organ dysfunction				Implicit ICD-10 coding abstraction of sepsis with organ dysfunction			
		Hospital admissions without agreement with validated study data, *n* = 373	Hospital admissions with agreement with validated study data, *n* = 625	OR (95%-CI)^*a*^	*p* value^*b*^	Hospital admissions without agreement with validated study data, *n* = 201	Hospital admissions with agreement with validated study data, *n* = 797	OR (95%-CI)^a^	*p* value^b^
Risk category					< 0.001				0.002
Previously healthy children	319	49 (15%)	270 (85%)	1.00		34 (11%)	285 (89%)	1.00	
Neonates	343	189 (55%)	154 (45%)	0.23 (0.13–0.4)		95 (28%)	248 (72%)	0.33 (0.18–0.63)	
Children with comorbidities	336	135 (40%)	201 (60%)	0.44 (0.26–0.73)		72 (21%)	264 (79%)	0.44 (0.25–0.79)	
Type of sepsis acquisition					0.2				0.04
Community-acquired	636	180 (28%)	456 (72%)	1.00		118 (19%)	518 (81%)	1.00	
Hospital-acquired	362	193 (53%)	169 (47%)	1.32 (0.89–1.96)		83 (23%)	279 (77%)	1.62 (1.02–2.57)	
Pathogens					0.001				0.1
*S. pneumoniae*	99	18 (18%)	81 (82%)	1.00		13 (13%)	86 (87%)	1.00	
*S. aureus*	147	42 (29%)	105 (71%)	0.75 (0.35–1.6)		20 (14%)	127 (86%)	0.91 (0.37–2.22)	
Coagulase-negative staphylococci	136	97 (71%)	39 (29%)	0.3 (0.14–0.68)		46 (34%)	90 (66%)	0.45 (0.18–1.09)	
*E. coli*	193	61 (32%)	132 (68%)	0.75 (0.35–1.6)		38 (20%)	155 (80%)	0.72 (0.31–1.69)	
*S. agalactiae*	76	20 (26%)	56 (74%)	1.62 (0.69–3.8)		16 (21%)	60 (79%)	1.25 (0.49–3.18)	
*Enterococcus* spp.	28	15 (54%)	13 (46%)	0.39 (0.13–1.13)		9 (32%)	19 (68%)	0.38 (0.12–1.18)	
*Klebsiella* spp.	46	18 (39%)	28 (61%)	0.86 (0.35–2.13)		5 (11%)	41 (89%)	1.79 (0.53–6)	
*N. meningitidis*	24	7 (29%)	17 (71%)	0.6 (0.2–1.79)		6 (25%)	18 (75%)	0.55 (0.17–1.8)	
*S. pyogenes*	49	11 (22%)	38 (78%)	0.48 (0.19–1.23)		5 (10%)	44 (90%)	0.89 (0.27–2.92)	
Viridans group streptococci	54	22 (41%)	32 (59%)	0.57 (0.24–1.33)		14 (26%)	40 (74%)	0.62 (0.24–1.62)	
Other pathogens	146	62 (42%)	84 (58%)	0.56 (0.27–1.16)		29 (20%)	117 (80%)	0.81 (0.35–1.88)	
Site or type of infection					< 0.001				0.1
Primary bloodstream	191	75 (39%)	116 (61%)	1.00		48 (25%)	143 (75%)	1.00	
Central line-associated bloodstream	273	162 (59%)	111 (41%)	0.49 (0.3–0.79)		70 (26%)	203 (74%)	1.05 (0.62–1.77)	
Urinary tract	107	16 (15%)	91 (85%)	2.27 (1.12–4.6)		12 (11%)	95 (89%)	2.32 (1.04–5.2)	
Pneumonia	95	23 (24%)	72 (76%)	0.96 (0.49–1.87)		11 (12%)	84 (88%)	1.41 (0.63–3.18)	
Central nervous system	76	26 (34%)	50 (66%)	0.68 (0.35–1.31)		20 (26%)	56 (74%)	0.64 (0.32–1.3)	
Gastrointestinal system	58	26 (45%)	32 (55%)	0.71 (0.36–1.38)		12 (21%)	46 (79%)	1.17 (0.54–2.53)	
Osteoarticular	60	3 (5%)	57 (95%)	4.8 (1.33–17.5)		2 (3%)	58 (97%)	4.6 (0.99–21.7)	
Skin and soft tissue	52	13 (25%)	39 (75%)	1.34 (0.62–2.92)		8 (15%)	44 (85%)	1.39 (0.57–3.38)	
Other infection sites	86	29 (34%)	57 (66%)	0.77 (0.42–1.42)		18 (21%)	68 (79%)	0.87 (0.44–1.73)	
ICU admission					0.002				0.3
No	491	121 (25%)	370 (75%)	1.00		78 (16%)	413 (84%)	1.00	
Yes	507	252 (50%)	255 (50%)	0.57 (0.39–0.82)		123 (24%)	384 (76%)	0.79 (0.52–1.22)	
Death					0.02				0.003
Survived	931	347 (37%)	584 (63%)	1.00		193 (21%)	738 (79%)	1.00	
Died	67	26 (39%)	41 (61%)	1.94 (1.11–3.41)		8 (12%)	59 (88%)	2.98 (1.35–6.6)	

*CI* confidence interval, *ICD* International Statistical Classification of Diseases and Related Health Problems, *ICU* intensive care unit, *OR* odds ratio

^a^Odds ratios from multivariate binomial regression model using the admission hospital as a random effect

^b^*p* values based on likelihood ratio tests

A total of 426 (74%) admissions with research-confirmed sepsis with organ dysfunction had been assigned implicit ICD-10 codes for sepsis with organ dysfunction with an accuracy of 74% (95%-CI 72–77) (Table 2). The sensitivity of implicit ICD-10 coding abstraction of sepsis with organ dysfunction increased from 62% (95%-CI 53–70) in 2012 to 67% (95%-CI 59–74) in 2015 (*p* = 0.5). In hospital admissions assigned implicit ICD-10 codes for sepsis with organ dysfunction, the case fatality ratio was 14% (95%-CI 11–17) (58 of 426), although 12% (8 of 66) deaths in children with sepsis with organ dysfunction were not captured. The agreement of implicit ICD-10 coding abstraction of sepsis with organ dysfunction varied by risk category was better in children who did not survive and if sepsis was hospital-acquired (Table 3).

### Agreement of ICD-10 coding abstraction of specific organ dysfunctions with validated study data

Children suffered from septic shock in 215 (22%), from respiratory dysfunction in 377 (38%), from hematological dysfunction in 286 (29%), from central nervous system dysfunction in 144 (14%), from hepatic dysfunction in 98 (10%), and from renal dysfunction in 71 (7%) hospital admissions. The sensitivity of explicit ICD-10 codes for septic shock was 43% (95%-CI 37–50) while it was 54% (95%-CI 48–61) for implicit ICD-10 coding abstraction of cardiovascular dysfunction. The sensitivity of implicit ICD-10 coding abstraction was 27% (95%-CI 22–31) for respiratory dysfunction, 53% (95%-CI 47–59) for hematological dysfunction, 8% (95%-CI 4–13) for central nervous system dysfunction, 13% (95%-CI 8–22) for hepatic dysfunction, and 58% (95%-CI 46–69) for renal dysfunction. The number of organ dysfunctions present was poorly represented by implicit ICD-10 coding abstraction (Additional file 1: Fig. S2).

### Comparison of estimates of national sepsis incidence based on ICD-10 coding abstraction compared to validated study data

**Fig. 1:**
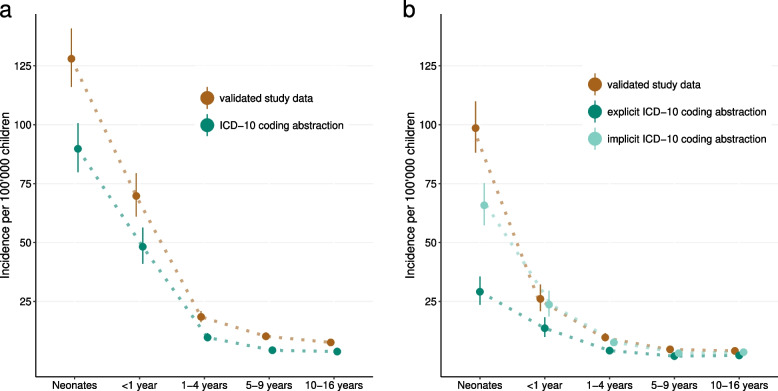
Comparison between using validated study data and ICD-10 coding abstraction to estimate the incidence of sepsis (**A**) and incidence of sepsis with organ dysfunction (**B**). Dots show the point estimates, and vertical lines represent the 95%-CIs around the point estimates for each age group. Estimates of the incidence of sepsis using validated study data are based on 989 hospital admissions, assuming participating hospitals captured 80% of admissions due to blood culture-proven sepsis in children less than 17 years of age in Switzerland (irrespective of age) [13]. Estimates of the incidence of sepsis using ICD-10 coding abstraction are based on 591 hospital admissions. Estimates of the incidence of sepsis with organ dysfunction using validated study data are based on 566 hospital admissions, assuming participating hospitals captured 90% of admissions due to blood culture-proven sepsis with organ dysfunction in children less than 17 years of age in Switzerland (irrespective of age) [13]. Estimates of the incidence of sepsis with organ dysfunction using explicit ICD-10 coding abstraction are based on 218 and for implicit ICD-10 coding abstraction on 420 hospital admissions

We calculated the incidence of sepsis using the 989 hospital admissions where sepsis occurred during the full study years (January 1, 2012, to December 31, 2015). The estimated national annual incidence of blood culture-proven bacterial sepsis was 21.0 per 100,000 children (95%-CI 19.8–22.2) based on validated study data, compared to 12.5 per 100,000 children (95%-CI 11.7–13.5) based on ICD-10 coding abstraction of sepsis. The estimated national incidence of blood culture-proven bacterial sepsis with any organ dysfunction was 12.0 per 100,000 children (95%-CI 11.1–12.9) based on validated study data, 4.6 per 100,000 children (95%-CI 4.1–5.2) based on explicit, and 8.9 per 100,000 children (95%-CI 8.2–9.7) based on implicit ICD-10 coding abstraction of sepsis with organ dysfunction. Overall, the incidence of sepsis was underestimated by ICD-10 coding abstraction across all age groups (Fig. 1).

## Discussion

In this population-based, multicenter, prospective cohort study on blood culture-proven sepsis in children, a comprehensive ICD-10 coding strategy only captured 60% of hospital admissions in children with sepsis and 66% of hospital admissions in children with sepsis with organ dysfunction. The sensitivity of explicit ICD-10 coding abstraction of sepsis with organ dysfunction was only 35%. Our findings show that ICD-10-based coding may severely underestimate sepsis burden, in terms of overall sepsis numbers, presence and severity of organ dysfunctions, and the number of children that die. Our results are consistent with previous reports of poor performance of ICD coding to capture sepsis [9–12, 23] which is concerning as most approaches to assess the burden of sepsis from a public health perspective remain exclusively based on coding-based databases.

Few studies have looked at the accuracy of ICD coding to identify pediatric patients with sepsis [11, 12]. In a single-center study in pediatric ICU patients with severe sepsis in the USA, explicit ICD-9 codes for severe sepsis (995.92) and septic shock (785.52) demonstrated a sensitivity of 72.8% and a PPV of 56.7% for the identification of patients with severe sepsis or septic shock, which is comparable to our findings on sepsis overall [11]. A multicenter study in children older than 60 days in 6 US pediatric centers found a sensitivity of 73% and a positive predictive value of 79% using the same ICD-9 coding strategy [12]. In contrast, explicit ICD-10 coding abstraction for sepsis with organ dysfunction had a sensitivity of 35% and a PPV of 90% in our cohort. We are not aware of any European cohort assessing coding accuracy in children with sepsis in the past 10 years.

The sensitivity of ICD-10 coding abstraction for sepsis varied by pathogen, site of infection, and the need for ICU admission. The agreement of explicit ICD-10 coding abstraction for sepsis with organ dysfunction varied by risk category, pathogen, site or type of infection, need for ICU admission, and survival. While the reasons underlying systematic underreporting of sepsis in coding are difficult to elicit, we suspect that clinicians are much more likely to classify patients according to the source of infection (e.g., “pneumonia”) and not necessarily as septic [24–26].

Even for cardiovascular dysfunction, which represents the only sepsis-associated organ dysfunction that can be coded specifically with the ICD-10 system, the sensitivity of explicit ICD-10 code was only 43%. While other organ dysfunctions associated with sepsis are not explicitly represented by the ICD-10 code, we found wide variations in the sensitivities of implicit ICD-10 coding abstraction for specific organ dysfunctions. This was especially accentuated in neonates, where the lack of agreed criteria and scoring systems for organ dysfunction [27] clashes with the fact that ICD-10 codes for neonates (P-Codes) are less versatile than other ICD-10 codes. ICD-10 codes for neonates are not used to annotate organ dysfunction because of sepsis.

There is some evidence that hospital claims for sepsis may be biased towards more severely ill patients [28]. This is supported in our study, as the 30-day case fatality ratio was 12% in hospital admissions with sepsis with organ dysfunction but increased to 18% in hospital admissions with explicit ICD-10 codes for sepsis with organ dysfunction. This is in contrast to a point prevalence study on 126 pediatric ICUs across 6 continents, where clinicians defined severe sepsis as less severe than sepsis defined by consensus definition, albeit that study did not include ICD codes [25]. Interestingly, only 60% of deaths in our dataset were captured by explicit ICD-10 codes, while implicit ICD-10 codes captured 87%. This may reflect that clinicians do not implicate sepsis as a contributor to death in children with complex medical conditions even if organ dysfunction and infection are associated with the demise of the patient [29].

ICD-11 [30], endorsed by WHO members in 2019, has addressed several of the shortcomings of ICD-10. Specifically, ICD-11 has implemented the Sepsis-3 definition of sepsis as a life-threatening organ dysfunction caused by a dysregulated host response to infection. ICD-11 allows for the capture of multi-organ dysfunction syndrome (ICD-11 MH16) and linking of information on the causative infectious agent and associated organ dysfunctions. Based on the low sensitivity of ICD-10 coding abstraction of sepsis with organ dysfunction compared to the validated study data, our findings suggest caution is warranted for future direct comparison of estimates of sepsis with organ dysfunction based on ICD-10 versus sepsis captured based on ICD-11.

Several limitations of this study need to be considered. First, the organization of the healthcare system in Switzerland and differences in demography and epidemiology of childhood sepsis may limit the generalizability of our findings to other settings. Second, organ dysfunctions captured by implicit ICD-10 codes for sepsis may reflect organ dysfunction resulting from other causes. Third, all children had blood culture-proven sepsis, preventing the calculation of specificity, accuracy, predictive values, and likelihood ratios of ICD coding abstraction of sepsis without organ dysfunction. Fourth, we could not assess the quality of ICD coding abstraction of sepsis due to viral infections or culture-negative sepsis. Given the over-representation of codes for bacterial infections in ICD-10, it is likely that we overestimated the accuracy of the ICD-10 abstraction of sepsis.

## Conclusions

We found a poor representation of sepsis with organ dysfunction by ICD-10 coding abstraction in children with bacterial sepsis in Switzerland. Based on our data, it is likely that studies assessing sepsis in children based on ICD-10 code abstraction severely underestimate the true burden of disease and that findings will not be comparable to estimates based on ICD-11. New definitions of sepsis in children should prioritize applicability to all clinical settings, to enable clinicians to correctly recognize and report sepsis, which will in turn improve medical coding.

## Supplementary Information

**Additional file 1: Supplementary Methods.** **Table S1.** Determinants of agreement of ICD-10 codes with pathogens detected in blood culture in neonates and children with sepsis. **Table S2.** Determinants of agreement of ICD-10 codes with prospective clinical diagnosis of sepsis in neonates and children. **Fig. S1.** Study flowchart. **Fig. S2.** Mosaic plot of the number of organ dysfunctions present according to implicit ICD-10 coding abstraction compared to validated study data 7. STARD Checklist.

## Data Availability

The data analyzed in this study are available from the corresponding author PKAA, pending approval of a methodological sound request by the study steering committee. The data are not publicly available because they contain information that could compromise research participant privacy.
