# Supplementary material for: Sensitivity of ICD coding for sepsis in children—a population-based study
Source: Intensive Care Med Paediatr Neonatal. 2023 Jun 13;1(1):5. doi: 10.1007/s44253-023-00006-1 (PMC10261837; doi:10.1007/s44253-023-00006-1)
Supplement: Supplementary file 1 — Additional file 1: Supplementary Methods. Table S1. Determinants of agreement of ICD-10 codes with pathogens detected in blood culture in neonates and children with sepsis. Table S2. Determinants of agreement of ICD-10 codes with prospective clinical diagnosis of sepsis in neonates and children. Fig. S1. Study flowchart. Fig. S2. Mosaic plot of the number of organ dysfunctions present according to implicit ICD-10 coding abstraction compared to validated study data 7. STARD Checklist. [file 44253_2023_6_MOESM1_ESM.pdf]

Supplementary Information

Sensitivity of ICD coding for sepsis in children - a population-based study

Authors

Olga Endrich, Karen Triep, Luregn J Schlapbach, Klara M Posfay-Barbe, Ulrich Heininger, Eric Giannoni, Martin Stocker, Anita Niederer-Loher, Christian R Kahlert, Giancarlo Natalucci, Christa Relly, Thomas Riedel, Christoph Aebi, Christoph Berger, and Philipp KA Agyeman on behalf of the Swiss Pediatric Sepsis Study

Correspondance

Philipp KA Agyeman, Department of Pediatrics, Inselspital, Bern University Hospital, University of Bern, Switzerland, philipp.agyeman@insel.ch

Contents

Supplementary Methods 2

Supplementary Table 1: Determinants of agreement of ICD-10 codes with pathogens detected in blood culture in neonates and children with sepsis 4

Supplementary Table 2: Determinants of agreement of ICD-10 codes with prospective clinical diagnosis of sepsis in neonates and children 5

Supplementary Figure 1: Study flowchart 6

Supplementary Figure 2: Mosaic plot of the number of organ dysfunctions present according to implicit ICD-10 coding abstraction compared to validated study data 7

STARD Checklist 8

Supplementary Methods

Pathogen-specific ICD-10 codes for sepsis:
A021, A327, A391, A392, A394, A400, A401, A402, A403, A408, A409, A410, A411, A412, A413, A414, A4151, A4152, A4158, A418, A419, A427, B377, P360, P361, P362, P363, P364, P365, P368, P369

ICD-10 codes for sepsis:
Pathogen-specific ICD-10 codes for sepsis AND any of A483, R572, R650, R651

ICD-10 codes for sepsis with organ dysfunction (explicit codes):
Pathogen-specific ICD-10 codes for sepsis AND any of A483, R572, R651

ICD-10 codes for cardiovascular dysfunction (explicit codes):
Pathogen-specific ICD-10 codes for sepsis AND any of A483, R572

ICD-10 codes for cardiovascular dysfunction (implicit codes):
A483, I958, P290, R031, R570, R571, R572

ICD-10 codes for respiratory dysfunction (implicit codes):
J80, J9600, J9601, J9609, J9690, J9691, J9699, P285

ICD-10 codes for central nervous system dysfunction (implicit codes):
G931, G934, P914, P915, P916, P919, R400, R401, R402, R410

ICD-10 codes for renal dysfunction (implicit codes):
N170, N171, N172, N178, N179, N181, N182, N183, N184, N185, N189, N19, P960, R34

ICD-10 codes for hepatic dysfunction (implicit codes):
K720, K7271, K7272, K7273, K7274, K7279, K729, K769

ICD-10 codes for hematologic dysfunction (implicit codes):
D618, D618, D619, D619, D6952, D6953, D6957, D6958, D6959, D6960, D6961, P60, P610, P616

ICD-10 codes for bacterial or fungal infection:
A010, A021, A327, A390, A391, A392, A393, A394, A395, A398, A399, A400, A401, A402, A403, A408, A409, A410, A411, A412, A413, A414, A415, A4151, A4152, A4158, A418, A419, A427, A490, A491, A492, A493, A498, A499, B370, B371, B372, B373, B374, B375, B376, B377, B378, B379, B950, B951, B952, B953, B9541, B9542, B9548, B955, B956, B957, B9590, B9591, B962, B963, B965, B966, B967, B968, P360, P361, P362, P363, P364, P365, P368, P369, P375

ICD-10 codes for (sepsis with) organ dysfunction (implicit codes):
Explicit ICD-10 codes for sepsis with organ dysfunction OR (ICD-10 codes for bacterial or fungal infection AND (implicit ICD-10 code for cardiovascular dysfunction OR respiratory dysfunction OR central nervous system dysfunction OR renal dysfunction OR hepatic dysfunction OR hematologic dysfunction))

ICD-10 codes for bacteremia:
A499

ICD-10 codes for S. aureus:
A410, B956, P362

ICD-10 codes for S. pneumoniae:
A403, B953, P361

ICD-10 codes for CONS:
A411, A412, B957, P363

ICD-10 codes for viridans group streptococci:
A408, A409, B9541, B9542, B9548, B955, P361

ICD-10 codes for group A streptococci:
A400, B950, P361

ICD-10 codes for group B streptococci:
A401, B951, P360

ICD-10 codes for Enterococcus spp:
A402, B952, P361

ICD-10 codes for other Gram-positive bacteria:
A412, A414, A418, B9590, B9591, B967, B968, P365, P368, P369

ICD-10 codes for E. coli:
A4151, B962, P364

ICD-10 codes for H. influenzae:
A413, B963, P368

ICD-10 codes for K. pneumoniae:
A4158, B962, P364

ICD-10 codes for N. meningitidis:
A390, A392, A393, A394, A395, A398, A399

ICD-10 codes for P. aeruginosa:
A4152, B965

ICD-10 codes for other Gram negative bacteria:
A010, A021, A414, A415, A4158, A418, B962, B963, B965, B966, B968, P365, P368, P369

ICD-10 codes for C. albicans:
B370, B371, B372, B373, B374, B375, B376, B377, B377, B378, B379, P375

Supplementary Table 1: Determinants of agreement of ICD-10 codes with pathogens detected in blood culture in neonates and children with sepsis

	All hospital admissions, n = 998	Agreement of ICD-10 coding abstraction of pathogens in sepsis with validated study data					
		No, n = 166		Yes, n = 832			
	n	n	%	n	%	OR[a] (95%-CI)	p value[b]
Risk category							0.8
Previously healthy children	319	52	16	267	84	1.00	
Neonates	343	54	16	289	84	0.87 (0.44-1.72)	
Children with comorbidities	336	60	18	276	82	0.84 (0.47-1.5)	
Type of sepsis acquisition							0.2
Community-acquired	636	97	15	539	85	1.00	
Hospital-acquired	362	69	19	293	81	0.71 (0.42-1.2)	
Pathogens							< 0.001
S. pneumoniae	99	25	25	74	75	1.00	
S. aureus	147	21	14	126	86	1.98 (0.86-4.5)	
Coagulase-negative staphylococci	136	38	28	98	72	0.62 (0.26-1.49)	
E. coli	193	9	5	184	95	3.83 (1.41-10.4)	
S. agalactiae	76	10	13	66	87	2 (0.75-5.3)	
Enterococcus spp.	28	6	21	22	79	0.77 (0.23-2.53)	
Klebsiella spp.	46	2	4	44	96	6.2 (1.27-30.5)	
N. meningitidis	24	1	4	23	96	3.41 (0.4-28.9)	
S. pyogenes	49	8	16	41	84	1.59 (0.57-4.4)	
Viridans group streptococci	54	9	17	45	83	1.46 (0.55-3.89)	
Other pathogens	146	37	25	109	75	0.8 (0.37-1.72)	
Site or type of infection							0.1
Primary bloodstream	191	35	18	156	82	1.00	
Central line-associated bloodstream	273	46	17	227	83	1.63 (0.9-2.98)	
Urinary tract	107	5	5	102	95	3.41 (1.12-10.4)	
Pneumonia	95	27	28	68	72	0.65 (0.31-1.38)	
Central nervous system	76	5	7	71	93	2.23 (0.75-6.6)	
Gastrointestinal system	58	11	19	47	81	1.11 (0.48-2.59)	
Osteoarticular	60	11	18	49	82	0.99 (0.38-2.57)	
Skin and soft tissue	52	12	23	40	77	0.91 (0.39-2.12)	
Other infection sites	86	14	16	72	84	1.44 (0.67-3.07)	
ICU admission							0.01
No	491	95	19	396	81	1.00	
Yes	507	71	14	436	86	1.9 (1.13-3.2)	
Presence of organ dysfunction							0.5
No	426	80	19	346	81	1.00	
Yes	572	86	15	486	85	1.19 (0.74-1.91)	
Death							0.4
Survived	931	158	17	773	83	1.00	
Died	67	8	12	59	88	1.42 (0.62-3.24)	

CI: confidence interval; ICD: International Statistical Classification of Diseases and Related Health Problems; OR: odds ratio; ICU intensive care unit.
[a] *Odds ratios from multivariate binomial regression model using the admission hospital as a random effect.*
[b] *p values based on likelihood ratio tests.*

Supplementary Table 2: Determinants of agreement of ICD-10 codes with prospective clinical diagnosis of sepsis in neonates and children

	All hospital admissions, n = 998	Agreement of ICD-10 coding abstraction of sepsis with validated study data					
		No, n = 402		Yes, n = 596			
	n	n	%	n	%	OR[a] (95%-CI)	p value[b]
Risk category							0.6
Previously healthy children	319	150	47	169	53	1.00	
Neonates	343	103	30	240	70	1.07 (0.63-1.81)	
Children with comorbidities	336	149	44	187	56	1.23 (0.77-1.97)	
Type of sepsis acquisition							0.4
Community-acquired	636	269	42	367	58	1.00	
Hospital-acquired	362	133	37	229	63	1.21 (0.79-1.84)	
Pathogens							< 0.001
S. pneumoniae	99	45	45	54	55	1.00	
S. aureus	147	71	48	76	52	1.12 (0.56-2.25)	
Coagulase-negative staphylococci	136	74	54	62	46	0.38 (0.18-0.81)	
E. coli	193	43	22	150	78	1.73 (0.84-3.57)	
S. agalactiae	76	13	17	63	83	3.47 (1.49-8.1)	
Enterococcus spp.	28	9	32	19	68	0.86 (0.31-2.44)	
Klebsiella spp.	46	17	37	29	63	0.81 (0.33-1.95)	
N. meningitidis	24	4	17	20	83	2.4 (0.69-8.4)	
S. pyogenes	49	28	57	21	43	0.79 (0.33-1.86)	
Viridans group streptococci	54	33	61	21	39	0.58 (0.26-1.31)	
Other pathogens	146	65	45	81	55	0.82 (0.42-1.61)	
Site or type of infection							< 0.001
Primary bloodstream	191	60	31	131	69	1.00	
Central line-associated bloodstream	273	124	45	149	55	0.82 (0.49-1.35)	
Urinary tract	107	22	21	85	79	2.58 (1.27-5.2)	
Pneumonia	95	40	42	55	58	0.83 (0.43-1.58)	
Central nervous system	76	18	24	58	76	0.99 (0.48-2.06)	
Gastrointestinal system	58	23	40	35	60	0.81 (0.4-1.64)	
Osteoarticular	60	50	83	10	17	0.19 (0.08-0.47)	
Skin and soft tissue	52	24	46	28	54	0.89 (0.42-1.89)	
Other infection sites	86	41	48	45	52	0.73 (0.39-1.35)	
ICU admission							< 0.001
No	491	262	53	229	47	1.00	
Yes	507	140	28	367	72	3.48 (2.28-5.3)	
Presence of organ dysfunction							0.5
No	426	209	49	217	51	1.00	
Yes	572	193	34	379	66	1.16 (0.79-1.7)	
Death							0.5
Survived	931	385	41	546	59	1.00	
Died	67	17	25	50	75	1.22 (0.64-2.32)	

CI: confidence interval; ICD: International Statistical Classification of Diseases and Related Health Problems; OR: odds ratio; ICU intensive care unit.
[a] *Odds ratios from multivariate binomial regression model using the admission hospital as a random effect.*
[b] *p values based on likelihood ratio tests.*

Supplementary Figure 1: Study flowchart

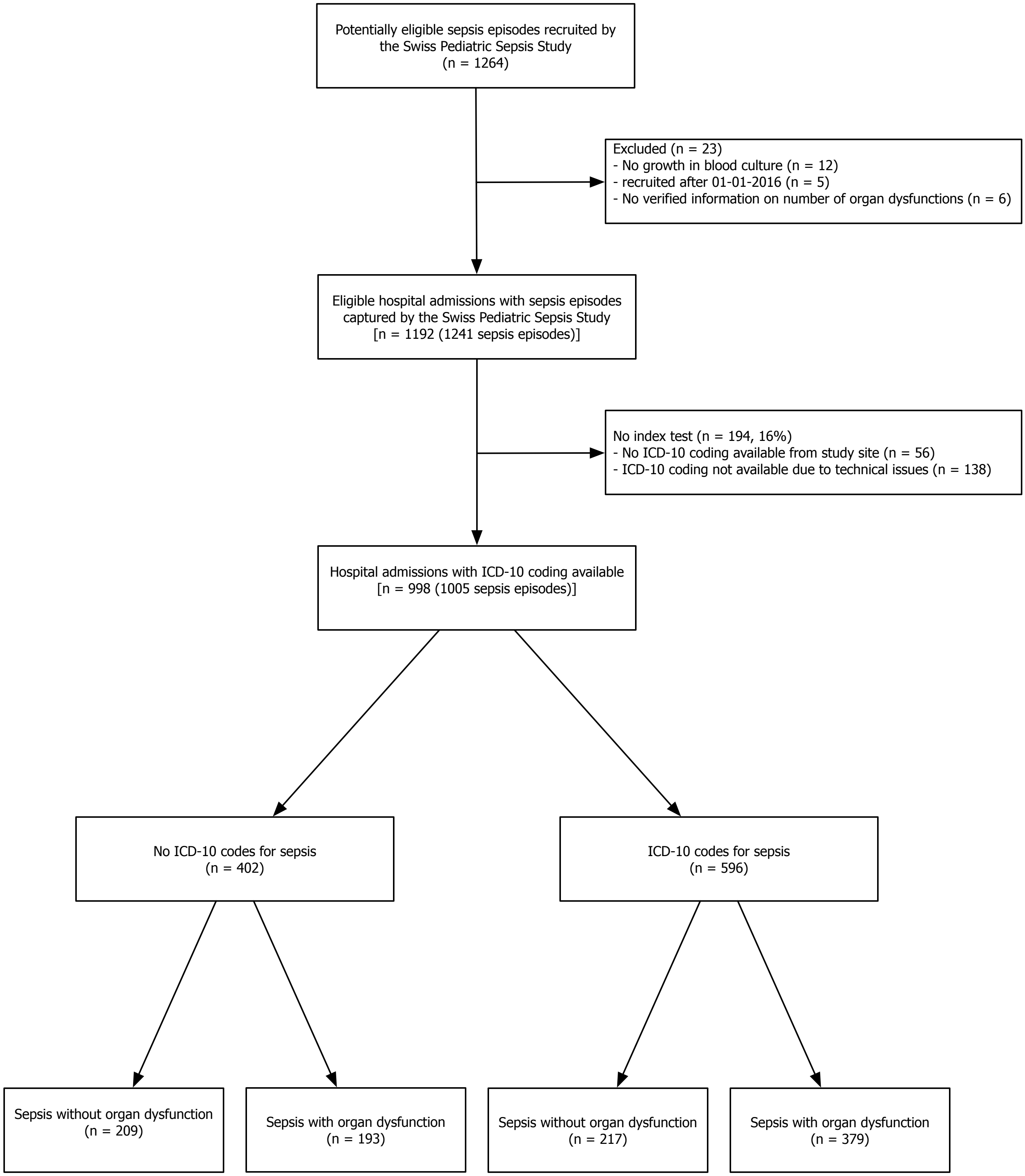

Supplementary Figure 2: Mosaic plot of the number of organ dysfunctions present according to implicit ICD-10 coding abstraction compared to validated study data

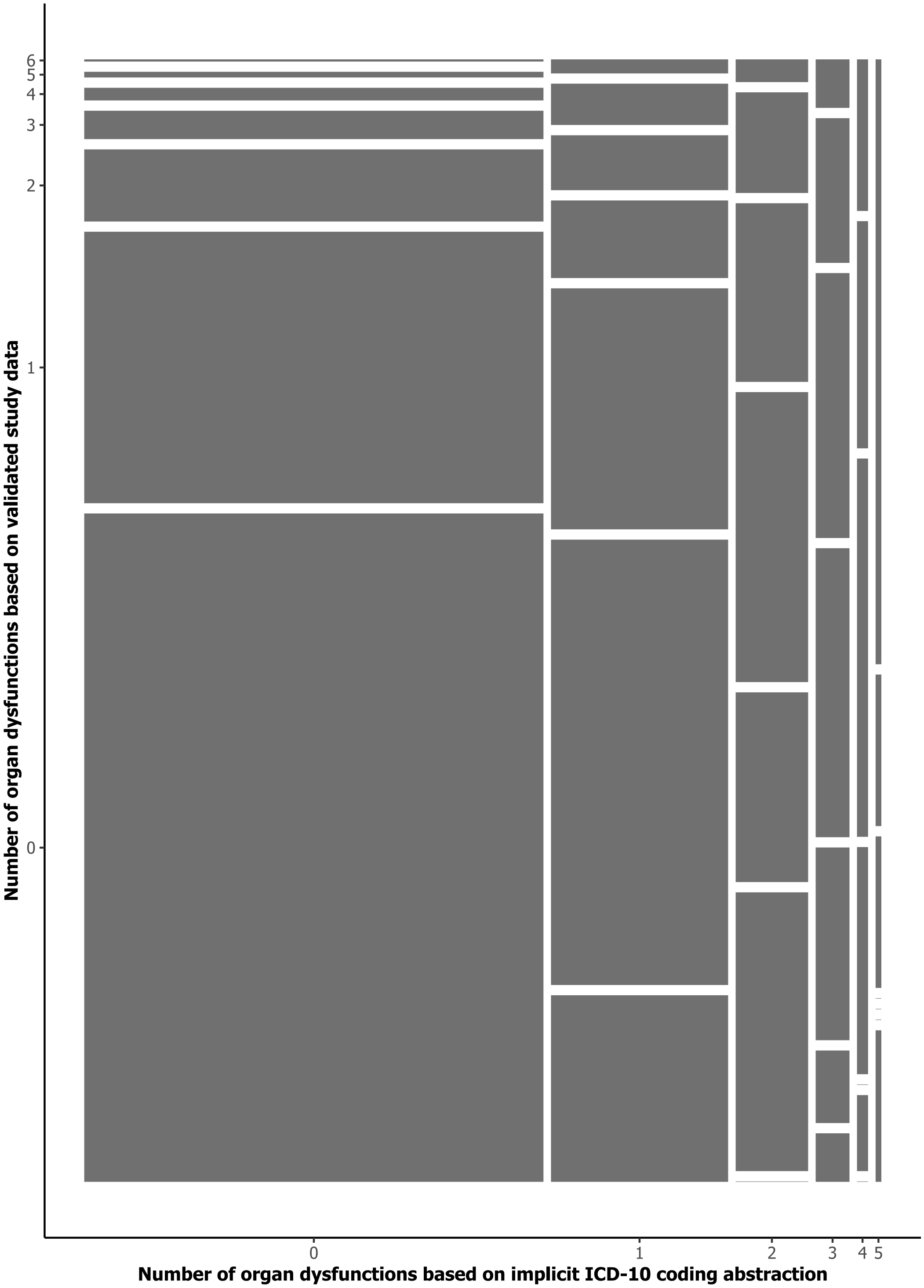

The y-axis shows the number of organ dysfunctions present according to validated study data and the height of each rectangle represents the proportion of admissions with the respective number of organ dysfunctions according to validated study data. The x-axis shows the number of organ dysfunctions present according to ICD-10 coding abstraction and the width of each rectangle represents the percentage of admissions with the respective number of organ dysfunctions according to implicit ICD-10 coding abstraction.

STARD Checklist

Section & Topic	No	Item	Reported on page #
**TITLE OR ABSTRACT**			
	**1**	Identification as a study of diagnostic accuracy using at least one measure of accuracy (such as sensitivity, specificity, predictive values, or AUC)	1
**ABSTRACT**			
	**2**	Structured summary of study design, methods, results, and conclusions (for specific guidance, see STARD for Abstracts)	2-3
**INTRODUCTION**			
	**3**	Scientific and clinical background, including the intended use and clinical role of the index test	5
	**4**	Study objectives and hypotheses	5
**METHODS**			
Study design	**5**	Whether data collection was planned before the index test and reference standard were performed (prospective study) or after (retrospective study)	6
Participants	**6**	Eligibility criteria	6-7
	**7**	On what basis potentially eligible participants were identified (such as symptoms, results from previous tests, inclusion in registry)	6-7
	**8**	Where and when potentially eligible participants were identified (setting, location and dates)	6
	**9**	Whether participants formed a consecutive, random or convenience series	6
Test methods	**10a**	Index test, in sufficient detail to allow replication	7 & supplementary information p2-3
	**10b**	Reference standard, in sufficient detail to allow replication	7
	**11**	Rationale for choosing the reference standard (if alternatives exist)	N/A
	**12a**	Definition of and rationale for test positivity cut-offs or result categories of the index test, distinguishing pre-specified from exploratory	N/A
	**12b**	Definition of and rationale for test positivity cut-offs or result categories of the reference standard, distinguishing pre-specified from exploratory	N/A
	**13a**	Whether clinical information and reference standard results were available to the performers/readers of the index test	6-7
	**13b**	Whether clinical information and index test results were available to the assessors of the reference standard	6-7
Analysis	**14**	Methods for estimating or comparing measures of diagnostic accuracy	8
	**15**	How indeterminate index test or reference standard results were handled	N/A
	**16**	How missing data on the index test and reference standard were handled	6
	**17**	Any analyses of variability in diagnostic accuracy, distinguishing pre-specified from exploratory	8
	**18**	Intended sample size and how it was determined	N/A
**RESULTS**			
Participants	**19**	Flow of participants, using a diagram	Supplementary Figure 1
	**20**	Baseline demographic and clinical characteristics of participants	9, Table 1
	**21a**	Distribution of severity of disease in those with the target condition	9, Table 1
	**21b**	Distribution of alternative diagnoses in those without the target condition	N/A
	**22**	Time interval and any clinical interventions between index test and reference standard	N/A
Test results	**23**	Cross tabulation of the index test results (or their distribution) by the results of the reference standard	9-11, Table 2
	**24**	Estimates of diagnostic accuracy and their precision (such as 95% confidence intervals)	9-11, Table 2
	**25**	Any adverse events from performing the index test or the reference standard	N/A
**DISCUSSION**			
	**26**	Study limitations, including sources of potential bias, statistical uncertainty, and generalisability	14
	**27**	Implications for practice, including the intended use and clinical role of the index test	12-14
**OTHER INFORMATION**			

	**28**	Registration number and name of registry	N/A
	**29**	Where the full study protocol can be accessed	N/A
	**30**	Sources of funding and other support; role of funders	3-4
